# The Minimal Domain of Adipose Triglyceride Lipase (ATGL) Ranges until Leucine 254 and Can Be Activated and Inhibited by CGI-58 and G0S2, Respectively

**DOI:** 10.1371/journal.pone.0026349

**Published:** 2011-10-19

**Authors:** Irina Cornaciu, Andras Boeszoermenyi, Hanna Lindermuth, Harald M. Nagy, Ines K. Cerk, Catharina Ebner, Barbara Salzburger, Astrid Gruber, Martina Schweiger, Rudolf Zechner, Achim Lass, Robert Zimmermann, Monika Oberer

**Affiliations:** Institute of Molecular Biosciences, University of Graz, Graz, Austria; National Institute for Medical Research, Medical Research Council, United Kingdom

## Abstract

Adipose triglyceride lipase (ATGL) is the rate-limiting enzyme of lipolysis. ATGL specifically hydrolyzes triacylglycerols (TGs), thereby generating diacylglycerols and free fatty acids. ATGL's enzymatic activity is co-activated by the protein comparative gene identification-58 (CGI-58) and inhibited by the protein G0/G1 switch gene 2 (G0S2). The enzyme is predicted to act through a catalytic dyad (Ser47, Asp166) located within the conserved patatin domain (Ile10-Leu178). Yet, neither an experimentally determined 3D structure nor a model of ATGL is currently available, which would help to understand how CGI-58 and G0S2 modulate ATGL's activity. In this study we determined the minimal active domain of ATGL. This minimal fragment of ATGL could still be activated and inhibited by CGI-58 and G0S2, respectively. Furthermore, we show that this minimal domain is sufficient for protein-protein interaction of ATGL with its regulatory proteins. Based on these data, we generated a 3D homology model for the minimal domain. It strengthens our experimental finding that amino acids between Leu178 and Leu254 are essential for the formation of a stable protein domain related to the patatin fold. Our data provide insights into the structure-function relationship of ATGL and indicate higher structural similarities in the N-terminal halves of mammalian patatin-like phospholipase domain containing proteins, (PNPLA1, -2,- 3 and -5) than originally anticipated.

## Introduction

In most organisms, excess energy is stored in form of triacylglycerol (TG) in lipid droplets (LDs). During periods of increased energy demand, TG undergoes a hydrolytic process termed lipolysis which results in the release of free fatty acids (FAs) and glycerol as energy substrates. Lipolysis is carried out as a hydrolytic cascade of consecutive reactions catalyzed by different lipases [1], [2], [3], [4]. Adipose triglyceride lipase (ATGL) was shown to be the rate-limiting enzyme in this process [1]. It catalyzes the hydrolysis of TG into diacylglycerol (DG) and FA during basal and hormone stimulated lipolysis [1], [5], [6], [7], [8]. The enzyme was discovered independently by three different laboratories and is also known as PNPLA2 (patatin-like phospholipase domain containing-2), desnutrin, phospholipase A2ζ and transport secretion protein 2.2 [1], [5], [6]. Patients with mutations in the gene coding for ATGL develop neutral lipid storage disease with myopathy (NLSDM) which is characterized by systemic TG accumulation in multiple tissues and cardiomyopathy [4], [9], [10].

**Figure 1:**
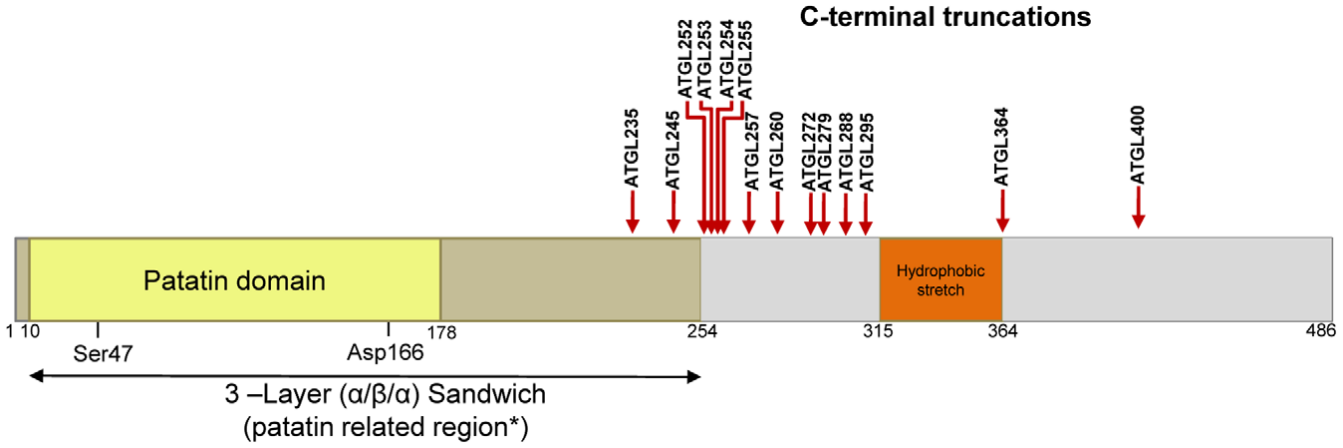
Domain organization of mouse ATGL and C-terminal truncations used in this study. Graphical representation of the predicted domain organization: in light yellow: patatin domain (residues 10–178), including the residues forming the proposed catalytic dyad (Ser47 and Asp166). Dark yellow: the 3-layer (α/β/α) sandwich (residues 10–254); Orange: the putative hydrophobic region. Red arrows indicate the 14 C-terminal deletions which were used in this study. *as shown in this work.

Mouse and human ATGL genes encode proteins with 486 and 504 amino acids, respectively and share 84% sequence identity. No 3D structure for ATGL is available; however sequence analysis revealed that ATGL harbors a patatin domain located between amino acids Ile10-Leu178 (Figure 1) [11]. In mammals, an entire protein family was classified as patatin-like phospholipase domain containing family (PNPLA) [12]. PNPLAs are characterized to different extents and are mostly lipid hydrolases with varying substrate specificities (e.g. TG, retinol ester, or phospholipid). Throughout all organisms, only two proteins with known 3D structures harbor the patatin domain: the name giving plant protein patatin, Pat17, and the catalytic domain of human cytosolic phospholipase A_2_ (cPLA_2_) [13], [14]. Based on similar features of ATGL with these proteins, it can be assumed that ATGL acts through a catalytic dyad similar to cPLA_2_ and Pat17 (Figure 1). The essential role of the putative dyad residues Ser47 and Asp166 in ATGL was experimentally confirmed by mutation studies [15], [16], [17]. *In vivo*, ATGL is reported to be localized in the cytoplasm, on LDs and in membranes [1], [18], [19]. LD localization is attributed to a hydrophobic stretch which is in the C-terminal half of ATGL, Val315 to Ile364 (Figure 1) [9], [20], [21]. Further indication that the catalytic activity of ATGL reside within the N-terminal portion of ATGL is based on analysis of TG hydrolase activity of C-terminally truncated proteins as found in patients with NLSDM. In living cells, these truncated ATGL variants are not capable of binding to lipid droplets and do not hydrolyze TG [15], [20], [21]. Interestingly, these truncated ATGL variants show increased TG hydrolase activity *in vitro* [15], [20], [21].

To date, our understanding of how ATGL activity is regulated is rather limited. Regulatory mechanisms include phosphorylation, protein-protein interaction and autoregulation by its own C-terminal domain [4], [16], [20], [22], [23], [24]. Very recently, residue Ser406 was shown to be phosphorylated by AMPK, which results in the activation of ATGL and enhances lipolysis [25]. A negative autoregulatory role of the C-terminal half of ATGL was suggested based on the observation that C-terminally truncated variants of ATGL showed even increased TG hydrolase activity *in vitro* as mentioned above [20], [21]. In an interesting study, Duncan *et al.* reported interaction between the N-terminal and C-terminal parts of ATGL, offering a possible explanation for this negative autoregulatory function [15].

Two proteins have been identified as important regulators for ATGL's TG hydrolase activity: comparative gene identification-58 (CGI-58, also known as Abhydrolase domain-containing protein 5, ABHD5) stimulates the activity of ATGL whereas the protein G0S2, encoded by the G(0)/G(1) switch gene 2, inhibits ATGL activity [16], [22], [26]. Direct protein-protein interactions between ATGL and full-length CGI-58, as well as N-terminally truncated variants of CGI-58 have been shown [16], [20], [22], [27]. Our group could also demonstrate previously, that ATGL stimulation also depends on the localization of CGI-58 to the LD since mutants of CGI-58, which were still able to interact with ATGL, failed to localize on the LD and failed to stimulate ATGL [27]. Other studies also showed CGI-58 mediated stimulation of ATGL variants, which were observed in NLSDM patients [20], [21], [28]. In 2010, G0S2 was identified as specific inhibitor of ATGL [22]. In the same study, the authors showed direct protein-protein interaction between G0S2 and an ATGL mutant lacking residues 259–337, which also leads to inhibition of ATGL activity. On the contrary, ATGL lacking the patatin-region (residue 10–178) did not interact with G0S2 [22]. G0S2 inhibited ATGL in a dose depended, non-competitive manner even in the presence of its co-activator CGI-58 [28].

Thus, data in the literature strongly suggest that stimulation of ATGL by CGI-58 and inhibition by G0S2 is mediated within the N-terminal half of ATGL. However, the exact requirements and a mechanistic explanation for its activation and inhibition are missing. Therefore, we investigated these important regulatory processes for ATGL activity. We determined the domain boundaries of ATGL which are responsible for exerting ATGL's TG hydrolyzing activity and its association in a systematic *in vitro* approach. Consistent with the existing data, our results show that C-terminal truncations of ATGL, expressed in *E. coli* exhibit increased activity and activation compared to the full length protein. We identified Leu254 as the domain boundary of ATGL, which is required for ATGL's TG hydrolase activity and its interaction with its co-activator CGI-58. Moreover, TG hydrolase activity of this minimal region of ATGL could be inhibited by G0S2. These findings indicate that the 254 N-terminal residues of ATGL are required and sufficient for TG hydrolysis and regulatory interactions with its co-activator CGI-58 and its inhibitor G0S2.

## Materials and Methods

### Cloning of recombinant proteins

Sequences containing the entire open reading frame of mouse ATGL (NCBI reference number AY894805), and mouse G0S2 (NCBI reference number NM_008059) were amplified using FailSafe™ PCR System (Epicentre Biotechnologies, Madison, USA) and Phusion Polymerase (New England Biolabs, Ipswich, USA), respectively. Used primers contained endonuclease cleavage sites for subsequent cloning procedures. PCR products and corresponding vectors were digested with the respective restriction enzymes. Full-length ATGL was cloned into a modified pET21a(+) (Novagen) vector, which carries a Gb1 sequence at the N-terminus, for increasing solubility of the fusion protein [29]. Mouse G0S2 was ligated into pCold DNA vector, with an N-terminal fusion tag, His_6_-trigger factor (TF) (Takara Bio Inc., Otsu, Japan). Shorter C-terminal constructs were produced using the QuickChange® Site-Directed Mutagenesis Kit (Agilent Technologies, USA) introducing a stop codon at the desired location. Used primers are listed in Table S1. Mouse ATGL 254 (ATGL254-MBP) was cloned also in a modified pMALc2x vector version E, comprising an uncleavable N-terminal MBP tag (supplied by Dr. Lars Pedersen, National Institute of Environmental Health Sciences, USA) [30]. All tags were tested as independent controls in further activity assays and did not exhibit any activities. The correct sequence of all inserts was confirmed by DNA sequencing analysis.

### Bacterial expression of recombinant proteins and preparation of cell extracts

*E.coli* strains BL21 and BL21(DE3)CodonPlus (Novagen) were grown in selective LB medium containing 50 µg/ml carbenicillin or 40 µg/ml kanamycin, respectively. Cultures of mouse ATGL254-MBP were supplemented with glucose (2 g/l). Expression of fusion proteins was induced with 0.5 mM IPTG for 3 hours or overnight at 15°C. For the preparation of cell extracts, cells were disrupted in buffer A (0.25 M sucrose, 1 mM ethylenediaminetetraacetic acid, 1 mM dithiothreitol, 20 µg/ml leupeptine, 2 µg/ml antipain, 1 µg/ml pepstatin, 50 µg/ml lysozyme pH 7.0) by sonication (Bandelin Sonoplus, HD2070, Berlin, Germany) on ice. Supernatants were collected after centrifugation at 21,000×g, 4°C for 20 min. Protein concentrations were determined using Bio-Rad protein assay (Bio-rad 785, Biorad Laboratories, GmbH, München, Germany).

### Expression of recombinant ATGL constructs in COS-7 cells

SV-40 transformed monkey kidney cells (COS-7, ATCC CRL-1651) were cultured in DMEM media (Invitrogen, Carlsbad, CA) with 10% fetal calf serum (Sigma-Aldrich, Taufkirchen, Germany) under standard conditions (95% humidified atmosphere, 37°C, 5% CO_2_). For transfections, cells (150.000/dish) were seeded in 6-well plates (Nunclone™, Nalge Nunc, Thermo Scientific, Rochester, USA) and incubated with 1 µg of pcDNA4/His Max plasmid coding for mouse ATGL (mATGL) or β-galactosidase (LacZ) which had been mixed with Metafectene (Biontex GmbH, München, Germany) as described [1]. Cells were harvested 2 days after transfection.

### Purification of mouse CGI-58

N-terminally His_6_-smt-tagged mouse CGI-58 was expressed as previously described [27]. Briefly, bacteria from overnight induction at 37°C were disrupted in 20 mM TrisHCl pH 7.8, 500 mM NaCl, 0.1% Igepal CA-630, 30 mM imidazole, 1 mM Tris(2-carboxyethyl)phosphine hydrochloride, 1 mM benzamidine, 100 µg/ml lysozyme and 0.1 mM phenylmethylsulfonyl fluoride. Soluble His-tagged fraction was purified by affinity chromatography using prepacked His-Trap FF 5 ml column (GE Healthcare, Waukesha, USA). The construct was eluted in 20 mM TrisHCl pH 7.8, 500 mM NaCl, 40% glycerol, 500 mM imidazole, 1 mM Tris(2-carboxyethyl)phosphine hydrochloride and 1 mM benzamidine.

### Purification of mouse ATGL254-MBP

After 6 h of expression at 15°C bacteria were lysed in 50 mM TrisHCl pH 7.4, 500 mM NaCl, 10% glycerol, 1 mM ethylenediaminetetraacetic acid, 1 mM Tris(2-carboxyethyl)phosphine hydrochloride, 1 mM benzamidine, 100 µg/ml lysozyme and 0.1 mM phenylmethylsulfonyl fluoride. For purification of target protein, the supernatant was incubated with amylose resin (New England Biolabs, Ipswich, USA) at 4°C, and eluted with 10 mM maltose.

### Western Blotting and quantitation

Expressions of ATGL, G0S2 and CGI-58 as fusion–proteins were confirmed by SDS-PAGE and/or Western blotting analysis using an anti-His primary antibody and anti-mouse HRP-linked secondary (GE Healthcare, Waukesha, USA). Chemoluminescence was induced using GE Amersham ECL Plus Kit and detected with a Typhoon 9400 Imager (GE Healthcare). Relative expression rates were determined using ImageQuant Software 5.2 (GE Healthcare) and the local median method for background correction.

### Assay for TG hydrolase activity

Different amounts of recombinant protein lysates (10–200 µg) were incubated in a final volume of 100 µl buffer A with 100 µl of substrate in a water bath for 60 min at 37°C. Substrate was prepared by emulsifying 0.33 µM triolein (Sigma, St. Louis, USA), 0.45 µM phosphatidylcholine/phosphatidylinositol (3∶1, Sigma, St. Louis, USA), 0.5% defatted bovine serum albumin (Carl Roth GmbH, Karlsruhe, Germany) and [9, 10-^3^H] triolein (10,000 cpm/µl; Perkin Elmer Life Sciences, Waltham, USA) as a radioactive tracer as described [1]. In control experiments, lysates of cells expressing only the N-terminal tags (Gb1, smt or Trigger factor -TF) were used. Reactions were terminated by addition of 3.25 ml of methanol/chloroform/heptane (10∶9∶7) and 1 ml of 0.1 M potassium carbonate, 0.1 M boric acid pH 10.5 and FAs extracted by vortexing. After centrifugation (800×g, 15 min), radioactivity in 1 ml of the upper phase was determined by liquid scintillation counting. Activation of ATGL was achieved by addition of purified CGI-58 (2.5 µg/100 µl reaction). Inhibition of ATGL in the presence of CGI-58 was tested by adding bacterial lysate containing G0S2.

Statistical significance was determined by Student's unpaired *t*-test (two tailed). Group differences were considered significant for p<0.05 (*), p<0.01 (**), and p<0.001 (***).

### Protein-protein interaction by ELISA

For the study of protein-protein interaction of truncated ATGL254 with CGI-58 and G0S2, ELISA plates (MaxiSorp, Nalge Nunc Int., Rochester, USA) were coated with 1 µg purified ATGL254-MBP or G0S2 in coating buffer (50 mM Tris-HCl, pH 7.4, 150 mM NaCl). Then wells were blocked with 5% BSA in coating buffer. In the next step 1–3 µg of purified His_6_-smt-tagged CGI-58 or MBP-tagged ATGL254 were used. As negative controls purified smt and MBP were verified throughout. After washing with coating buffer containing 0.05% Tween 20, mouse anti-his antibody (GE Healthcare), diluted 1∶500 in coating buffer containing 0.5% BSA was added for detection of His-tagged proteins. After three washes, horseradish peroxidase-conjugated anti-mouse antibody (GE Healthcare) was added at a dilution of 1∶1000. Alternatively, for the detection of ATGL254-MBP protein the plates were incubated with Anti-MBP-HRP antibody (New England Biolabs, Ipswich, USA). After three washes, tetramethyl-benzidine and H_2_O_2_ were added and the reaction stopped by addition of HCl. Absorbance was determined at 450 nm using 620 nm as reference wavelength.

### 3D Model of mouse ATGL

The Yasara software suite was used for homology modeling of the minimum active domain of ATGL. 3D coordinates of patatin 17 (Pat17, PDB code 1OXW, Ala23-Y316) and the catalytic domain of cPLA_2_ (PDB code 1CJY, residues Val187-Val641) were used as templates [31]. The sequence identities of ATGL254 were only 12% when aligned with Pat17 and 10% with cPLA_2_. The Z-scores of the model calculated by Yasara were −0.189 for dihedral angles, −3.037 and −2.891 for 1D and 3D packing, respectively. The quality and the geometry of the model was also assessed using MolProbity [32]. The all-atom clashscore (giving the number of serious steric overlaps per 1000 atoms) was 0.51. 92.9% and 98.8% of the residues were found in the favored and allowed regions of the Ramachandran plot, respectively. The same fold for ATGL ranging from residue 4–189 or 4–320 is also predicted in the Protein Model Portal [33]. The highest scoring models calculated using the Phyre server also used Pat17 and cPLA_2_ as templates [34]. These modeled regions (10–178 using Pat17, or 3–101 and 3–61 using cPLA_2_ as template) share the same overall fold as our model of ATGL254 in the identical regions. It should also be noted that both prediction servers also picked up short stretches with similarities to malonyl CoA-acyl carrier protein transacylase. The alignment of the model, Pat17 and the catalytic domain of cPLA2 was performed using lsqman [35].

## Results

### Activation and inhibition of ATGL can be studied in a simplified model system of lipolysis

In order to gain more insight into the basic mechanism and structural requirements of ATGL function, its stimulation by CGI-58 and inhibition by G0S2, we expanded a simplified experimental setup which was used before to mimic the first step of lipolysis [27]. Thereby, only TG hydrolysis *per se* is reconstituted with proteins expressed in *E.coli* and an artificial TG substrate. *In vitro* assays with mouse ATGL (mATGL) expressed in bacterial cultures harbors the advantages of eliminating possible unidentified factors responsible for reported cell-type dependence and the influence of eukaryotic proteins (e.g. LD associated proteins) from the complex equation of lipolysis.

**Figure 2:**
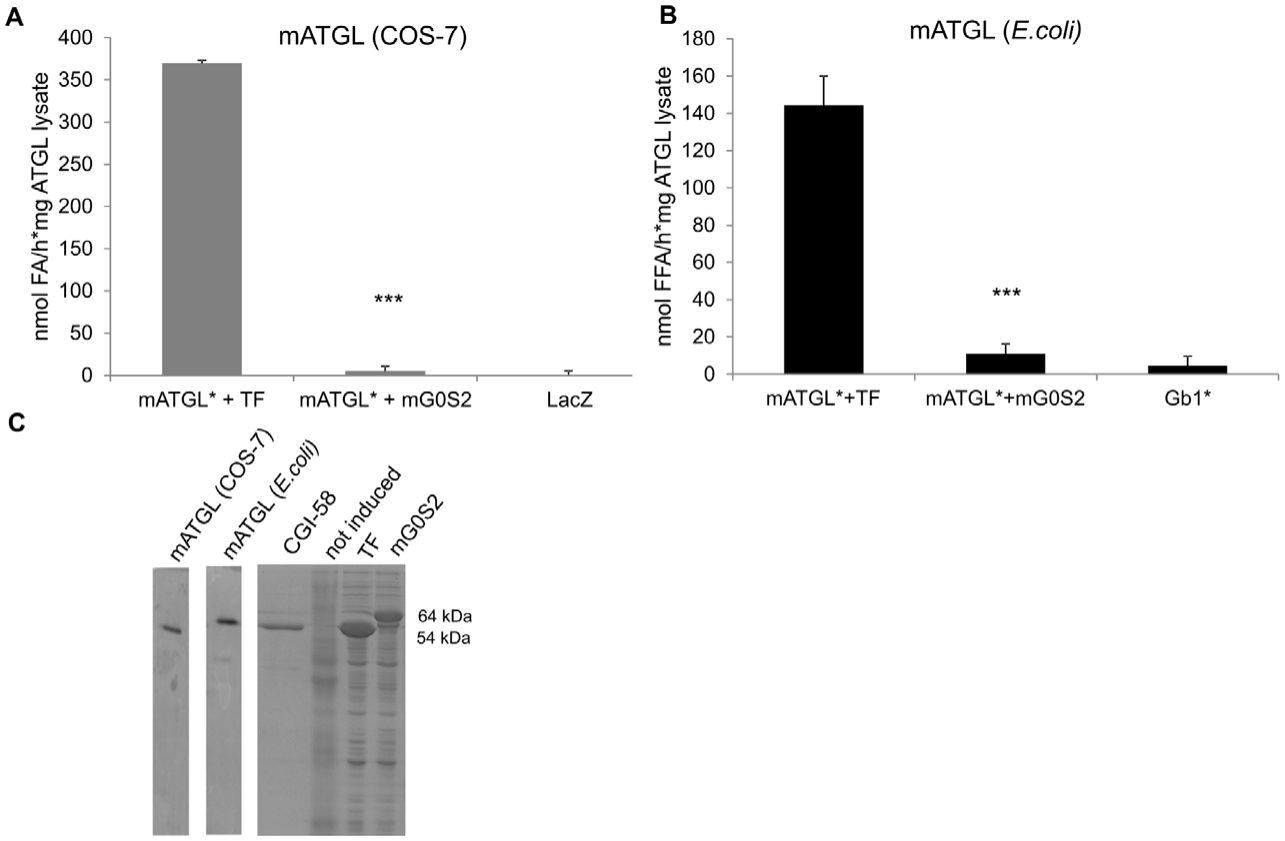
TG hydrolase activity of full length ATGL is inhibited by G0S2. **A.** Mouse ATGL (mATGL) contained in COS-7 cell lysates were subjected to *in vitro* TG hydrolase activity assay in the presence of CGI-58 (*) and without or with addition of bacterially expressed mouse G0S2 (mG0S2), using radiolabeled triolein as artificial substrate. **B.** *E. coli* expressed mATGL was assayed in *in vitro* TG hydrolase activity assay without or with mG0S2 as above. Gb1, the fusion tag of mATGL, does not exhibit TG hydrolase activity. Representative assays (performed in triplicates) of three independent experiments are shown. Data are presented as mean+SD. *** indicate statistical significant differences as determined by unpaired Student's *t*-test (two-tailed), *p*>0.001. As control, the corresponding fusion tag (Trigger factor –TF for mG0S2) was added to the reaction. **C.** Western blots confirming expression of mATGL in COS-7 cells and in *E.coli*. SDS-PAGE showing purified CGI-58 (∼54 kDa) and bacterial lysates of TF-mG0S2 (64 kDa), and TF alone (54 kDa). As a control, lysates of non induced cells were used.

Previously we had shown that the activity of mATGL can also be stimulated by mouse CGI-58 (mCGI-58) expressed in *E. coli* [27]. We first wanted to verify that bacterially expressed mouse G0S2 (mG0S2) is functional at inhibiting mATGL expressed in COS-7 cells after activation by CGI-58. Indeed, inhibition of mATGL expressed in COS-7 and also in *E.coli* was observed upon addition of mG0S2 from *E.coli* lysates in a similar manner (Figure 2A and B). This indicates, that the underlying protein-protein interaction is independent from post-translational modifications and can be studied at the level of heterologously expressed proteins.

In all our assays, mG0S2 was added as N-terminal His_6_-Trigger-factor (TF) fusion protein. This fused protein does not inhibit ATGL activity by itself and the effect of G0S2 was compared with ATGL activity/stimulation in presence of this fusion partner. Expression of the various proteins was confirmed by Western blotting analysis (for ATGL constructs) and SDS-PAGE (for the other recombinant proteins) (Figure 2C).

### ATGL's TG hydrolyzing activity is abrogated upon deletion of the C-terminal residues beyond Leu254

Next, we determined the minimal length of ATGL which is catalytically active in TG hydrolysis. Previous reports indicate that the hydrolase activity of ATGL is conferred by the N-terminal portions of the protein [20], [21]. To date, the shortest ATGL construct reported to be still active in hydrolyzing TG was an N-terminal region of the protein truncated at residue 289 [20].

In order to determine the minimal domain for ATGL activity, we generated 14 different C-terminally truncated ATGL variants, heterologously expressed them in *E coli*, and subjected lysates to hydrolase activity assay (Figure 1 and Table S1). We chose ATGL truncations based on the predicted overall domain organization, secondary structure, and sequence conservation within mammalian species. Sequence conservation between human and mouse ATGL showed a clear distinction between the highly conserved N-terminal half (residues 1–266, 92% sequence identity) and the less conserved C-terminal half (residues 267–486, 81% sequence identity).

**Figure 3:**
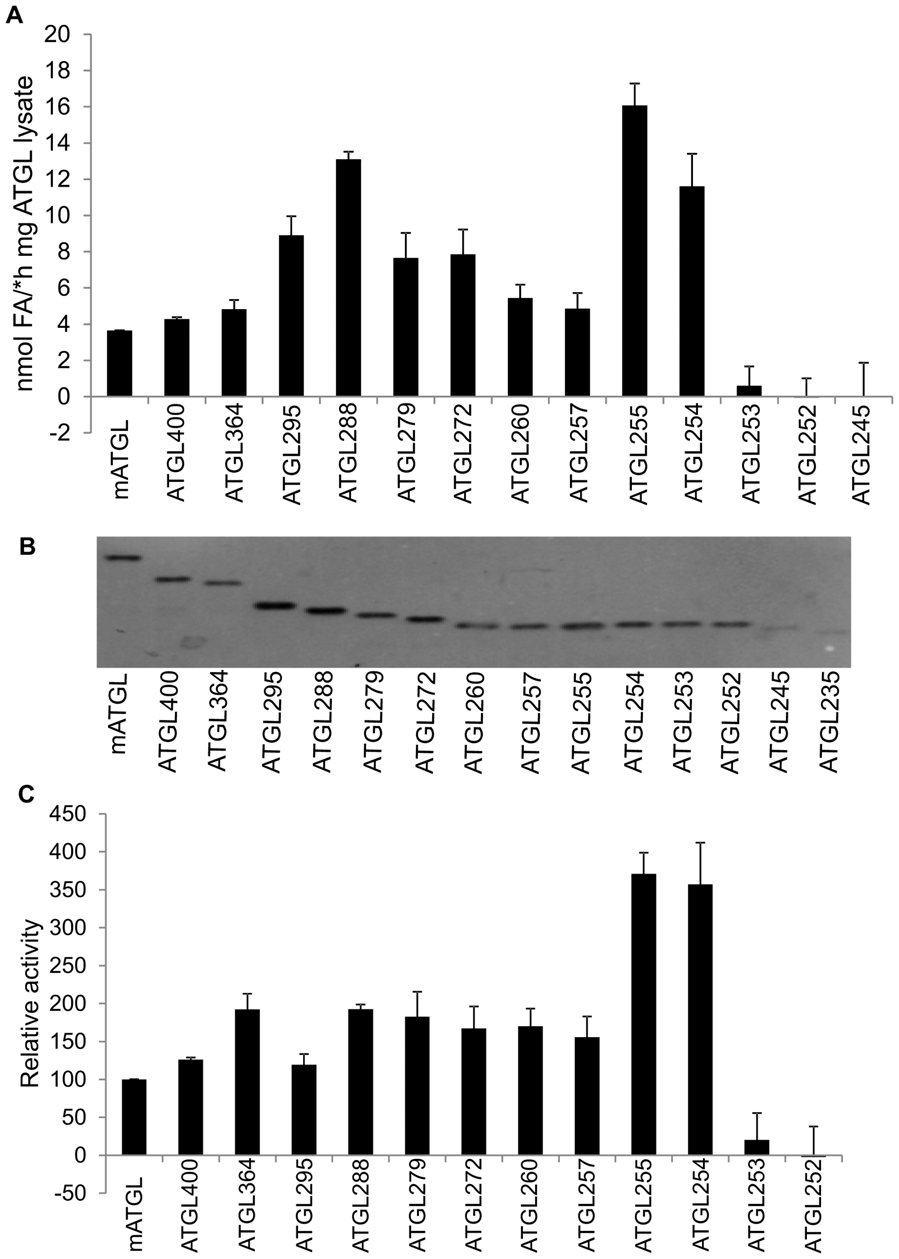
The N-terminal region of ATGL up to residue 255 is necessary for enzymatic activity. **A.** C-terminally truncated ATGL variants were expressed in *E.coli*, lysates prepared, and TG hydrolase activity determined *in vitro* using radiolabeled triolein as substrate. The determined activity was normalized to bacterial lysate protein contents. The experiment was performed in triplicates. A representative result of three independent experiments is shown. **B.** Expression was assessed by Western blotting and ATGL in the soluble fraction of the bacterial lysates was quantified by densitometric analysis. **C.** TG hydrolase activities obtained in A were normalized to expression levels of ATGL variants as assessed in B. Data are presented as mean+SD.

Results of TG hydrolase assays clearly demonstrated that truncated proteins of mATGL up to Leu254 (termed ATGL254 throughout this paper) retained the ability to hydrolyze TG (Figure 3A). In contrast, mATGL variants with truncations closer to the N-terminus, at residues 253, 252, 245 and 235, lost their TG hydrolyzing activity. Thus, we can conclude that ATGL254 represents the shortest fragment of ATGL which retains TG hydrolase activity (Figure 3A). Next, we correlated specific activities of truncated variants to full-length ATGL. The rational for this approach is that enzymatic activities measured in protein lysates obviously depend on the amount of the protein in the soluble fraction. In addition, this procedure allowed us to place these data in context with previous reports, which showed higher activities of C-terminally truncated ATGL variants [20]. Western Blot analysis confirmed the presence of soluble ATGL variants in the lysates. Differences in the expression and solubility levels were evident (Figure 3B). To account for these differences in expression/solubility levels, results of the Western Blot was analyzed densitometrically and used for calculating relative activity rates. These relative activities, as depicted in Figure 3C, clearly demonstrate that shorter fragments of ATGL have higher intrinsic *in vitro* TG hydrolyzing activity when compared to full-length ATGL. The activity of C-terminal ATGL truncations was ablated when shorter fragments than ATGL254 were tested (Figure 3C), in line with ATGL254 as the minimal fragment required for ATGL activity.

### CGI-58 stimulates C-terminal truncations of ATGL including ATGL254

**Figure 4:**
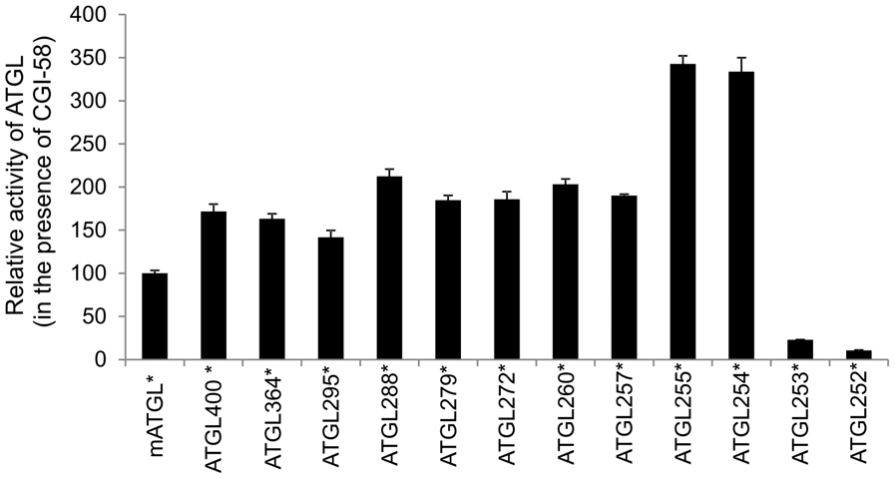
CGI-58 stimulates C-terminal truncation variants of ATGL including ATGL254. TG hydrolase assay with different C-terminal truncations of ATGL, in the presence (*) of purified CGI-58, was performed as described in Figure 2. TG hydrolase activities were normalized to that of full length ATGL ( = 100%). Data are presented as mean+SD. Results are representative of three independent experiments.

In the next experiments we asked whether shortened versions of ATGL can also be stimulated by addition of CGI-58. Therefore, we performed TG hydrolase experiments using full-length ATGL and C-terminally truncated variants in the presence of mCGI-58 and related measured activities to activated full-length ATGL levels. Interestingly, all active fragments of ATGL were stimulated by CGI-58 (Figure 4). Importantly, ATGL254 was also stimulated by CGI-58, suggesting that this fragment of ATGL is still capable of interacting with CGI-58. Consequently, C-terminal residues of ATGL beyond Leu254 are not essential for activation by CGI-58. Shorter constructs, such as ATGL253 and ATGL252, retained virtually no detectable TG hydrolase activity and were not stimulated to a significant extent by CGI-58 (∼77% and 90%, respectively, less than full length ATGL; Figure 4). As expected, all shorter constructs tested (ATGL245 and ATGL235) were inactive and could not be activated by CGI-58 (data not shown). These data clearly demonstrate that ATGL254 is the shortest fragment of mATGL that retains the ability to be activated efficiently by CGI-58.

### Shortest active fragment of mouse ATGL can also be inhibited by G0S2

**Figure 5:**
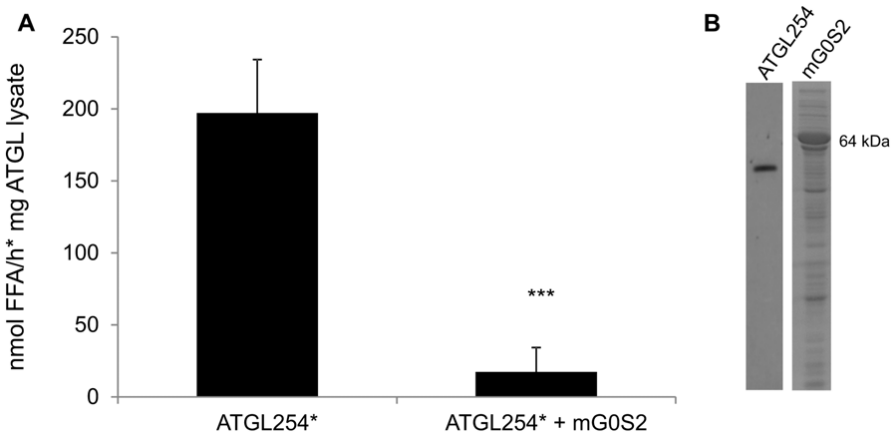
Minimal active fragment of ATGL can be inhibited by G0S2. **A.** ATGL254 was expressed in *E. coli*, lysates prepared, and TG hydrolase activity assay performed in the presence of purified CGI-58 (*), without and with addition of mG0S2 as described in Figure 2. Data are presented as mean+SD and representative for three independent experiments, each performed in triplicates. *** indicate statistical significant differences as determined by unpaired Student's *t*-test (two-tailed), *p*>0.001. **B.** Western Blotting analysis of ATGL254 expression and SDS-PAGE gel confirming expression of mG0S2 in bacterial lysate.

Next we asked whether ATGL254, the minimal active fragment of ATGL, can also be inhibited by G0S2. As shown in Figure 5, CGI-58 activated ATGL254 can be inhibited by addition of bacterial lysate of G0S2. This also suggests that the protein surfaces for interaction of ATGL with G0S2 reside within the first 254 residues of the protein, similar to the results observed for protein interaction with CGI-58.

### ATGL254 physically interacts with CGI-58 and G0S2

**Figure 6:**
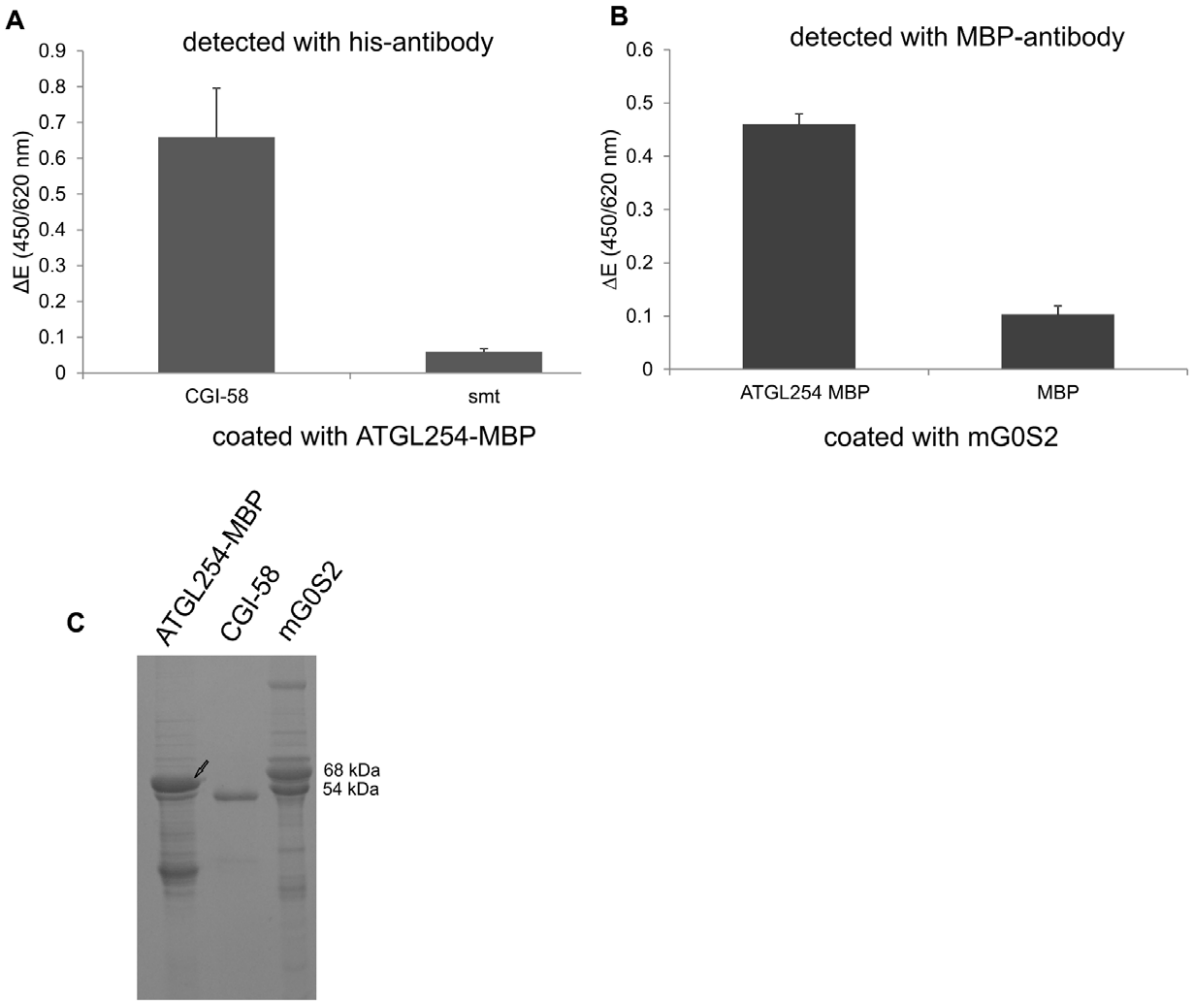
ELISA experiment demonstrating that ATGL254 interacts with CGI-58 and G0S2. **A.** ELISA plates were coated with purified ATGL254-MBP and then incubated with purified His-tagged CGI-58. Purified smt (the fusion tag for CGI-58) was used as a negative control. Detection was performed by anti-his-antibody, HRP-labeled secondary antibody and tetramethyl-benzidine as substrate. **B.** ELISA plates were coated with purified mG0S2 and then incubated with purified ATGL254-MBP. Purified MBP was used as negative control. Detection was performed using an anti-MBP (HRP conjugated) antibody and otherwise as in A. Data are presented as mean+SD and representative for 2 independent experiments (performed in triplicates). **C.** SDS-PAGE confirming the expression and purification of respective fusion proteins.

To further substantiate our conclusions that ATGL254 is sufficient for regulation by CGI-58 and G0S2, the implied physical interaction between the involved proteins was also determined by ELISA experiments. For this purpose, plates were coated with purified ATGL254 or mG0S2. Then, wells were incubated with purified His_6_-smt-tagged CGI-58 or maltose binding protein (MBP)-tagged ATGL254. As expected, protein-protein interactions between the ATGL254 and its co-activator CGI-58, as well as its inhibitor G0S2 were detected (Figure 6A and B). These data further support the conclusion that ATGL254 harbors all residues required for catalytic activity as well as interaction with ATGL's regulatory proteins, CGI-58 and G0S2.

### The 3D model of ATGL indicates that residues beyond amino acid Leu178 are involved in completing the patatin-related domain

**Figure 7:**
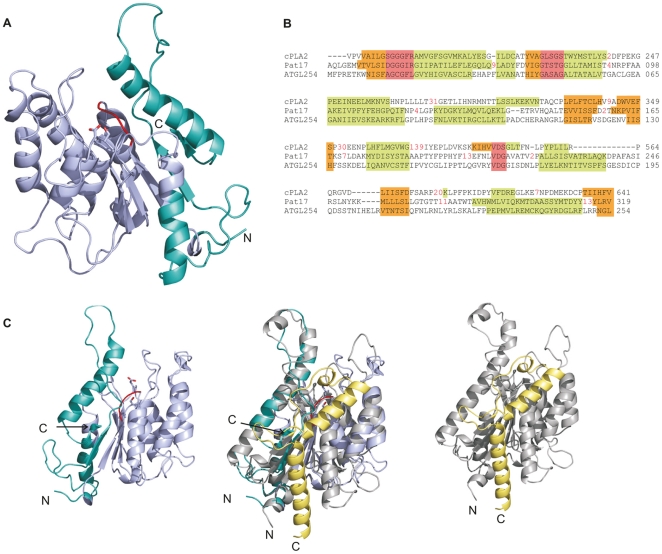
Model of mouse ATGL254. **A.** Homology-modeled structure of mouse ATGL ranging from residue Met1-Leu254 in cartoon representation. Residues Trp8-Leu178 are colored in light blue. Ser47 and Asp166, which form the catalytic dyad, are colored by atom; Gly14-Phe17, which are thought to be involved in forming the oxyanion hole are in red. N- and C-termini are indicated. **B.** Structural alignment of Pat17 (PDB code 1OXW) and cPLA_2_ (PDB code 1CJY) with the 3D model of ATGL254. Residues at the catalytic site (oxyanion hole, GXSXG motif, catalytic Asp) are highlighted in red. α-helices and β-strands are indicated in green and orange, respectively. Red numbers indicate extra amino acids in the structure of Pat17 and cPLA_2_. **C.** Left panel: model of ATGL254; middle panel: overlay of the 3D model of ATGL254 with Pat17 (colored grey and yellow); right panel: structure of Pat17. Val319-Lys383 of Pat17 (yellow) suggest a possible further structural organization of approximately 60 additional residues in mATGL.

After establishing the minimal requirements for retaining ATGL's enzymatic activity, we wanted to gain more insight into the structural background enabling the catalytic activity and its protein-protein interactions. Currently, no experimental 3D structure is available for ATGL. Therefore we performed homology modeling of ATGL ranging until residue Leu254. Pat17 and cPLA_2,_ the two patatin family members with known 3D structures, were used as templates. Due to the overall low sequence identity with the templates, the resulting models do not provide atomic details. Therefore it cannot be used for detailed interpretation of the structure, however should be viewed as giving a glimpse of the overall structure of the protein and its domain boundaries. Insights into the potential domain architecture of the N-terminal half of ATGL are given by the model (Figure 7). The template Pat17 is a 386 residue protein. Its crystal structure revealed a single-domain protein, harboring a central β-sheet with two α-helices on the concave side and 7 α-helices on the convex side. Although the patatin fold shares similarities with the canonical α/β hydrolase fold, it also harbors features that are clearly distinct from the α/β hydrolase fold, e.g. a six-stranded sheet as the core, in which five parallel sheets are followed by one anti-parallel strand, and the hydrolytic activity is carried out by a catalytic dyad [14]. In the 3D model of ATGL254, the catalytic dyad residues Ser47 and Asp166 along with a potential oxyanion hole forming residues Gly14-Phe17 are in an overall spatial arrangement which allows enzymatic activity. Residues Asn9-Leu178 in mATGL (blue in Figure 7A and C), which correspond to residues Thr30–Leu227 in Pat17, contribute to 4 of the 6 strands in the central β-sheet. The helical packing on the convex side of the central sheet of the ATGL model allows the formation of a compact arrangement of secondary structure elements. However, residues within this range are only involved in forming one partial α-helix at the concave side of the protein (Figure 7A and C, compare also with Figure 3 in [4]). Therefore it seems very likely, that these residues are not sufficient to build a stable and functional protein domain and that residues beyond Lys179 (cyan in Figure 7A and C) play an integral part in forming the active protein domain. Interestingly, residues Lys180 to Leu254 further complement the central parallel β-sheet and form helices on the concave side of the sheet in our model (colored cyan in Figure 7A). These residues are probably essential for a stable fold of ATGL and play a crucial role in creating the core of this protein domain. In summary, our 3D modeling data are in agreement with our biochemical observations, showing that residues within the N-terminal part of ATGL until Leu254 are essential for forming a stable, active protein domain.

## Discussion

ATGL is the rate-limiting enzyme of lipolysis and its enzymatic activity is enhanced by CGI-58 and inhibited by G0S2 [1], [16], [22]. Mechanistic explanations for ATGL's activation and inhibition are rather scarce. Mutagenesis studies confirm the importance of the proposed catalytic dyad residues Ser47 and Asp166 [15], [16], [17]. C-terminal truncations with premature stop-codons and frame shifts (Q289X, W367X, FS270, FS282) that retain TG-hydrolyzing capacity have also been reported [15], [20], [21]. Some of these truncations show an apparent increased *in vitro* catalytic activity and suggest a regulatory role for the C-terminal region of ATGL [15], [20], [21].

In the present study we determined the minimal domain requirements of ATGL for enzymatic activity and the basis for its interaction with CGI-58 and G0S2. In agreement with previous reports, we show that C-terminal truncations beyond residue 280 are functional in hydrolyzing TG. Our systematic approach to determine the minimal domain requirements for activity (Figure 3A) demonstrated that the loss of activity is very abrupt when comparing the activity of the truncations at residues Leu255, Leu254, and Gly253. C-terminal deletions up to Leu254 retain TG hydrolase activity, implying that the structural basis for enzymatic activity is also preserved. Possible explanations for the loss of activity of larger truncations are incorrect folding of the minimal domain and/or failure of substrate recognition.

The TG hydrolyzing activity of the tested ATGL constructs did not diminish gradually, on the contrary, higher levels of activities were observed for all C-terminally truncated fragments compared to full-length protein (Figures 3 and 4). Especially high activities in the presence and absence of CGI-58 (∼3.5 fold increase compared to full length ATGL) were observed for the last two active C-terminally truncated ATGL variants, ATGL255 and ATGL254. Such high activities could result from the previously reported inhibitory effect of C-terminal regions of the protein [20]. Interestingly, the shortest active ATGL fragment ATGL254 also retained the ability to be inhibited by G0S2 (Figure 5), indicating that the interaction surface of ATGL with its protein inhibitor is also located within the N-terminal half of the protein. This was further confirmed by protein-protein interaction studies demonstrating protein-protein interaction between ATGL254 and ATGL's regulatory proteins CGI-58 and G0S2 (Figure 6A and B). Nevertheless, additional modulation of protein-protein interaction of ATGL with CGI-58 and/or G0S2 by C-terminal residues of ATGL cannot be ruled out at this point.

The finding that mATGL variant ATGL254 is active in hydrolyzing TG and interacts with CGI-58 and G0S2, prompted us to generate a 3D structure model (Figure 7). The model provides first structural insights into the structure of the functionally active minimum domain of ATGL and an overall structural arrangement of the active site. It shows that the 3D structural elements of the patatin-related region (residues 8–178 (dark blue)) build a mostly parallel sheet with α-helices flanking one side of the protein (Figure 7A and C) [14]. However, the 3D structures of Pat17 and cPLA_2_ harbor additional β-strands in their central sheet and α-helices also pack at the other side of the protein. Our model of ATGL indicates that residues up to Leu254 might form the apparently missing components of the core structure by adding two helices on the concave face of the strand and being just sufficient to form a six-stranded central β-sheet (Figure 7). Therefore, we propose that the patatin-related region of ATGL stretches beyond residue Leu178 and also includes amino acids until residue Leu254.

**Figure 8:**
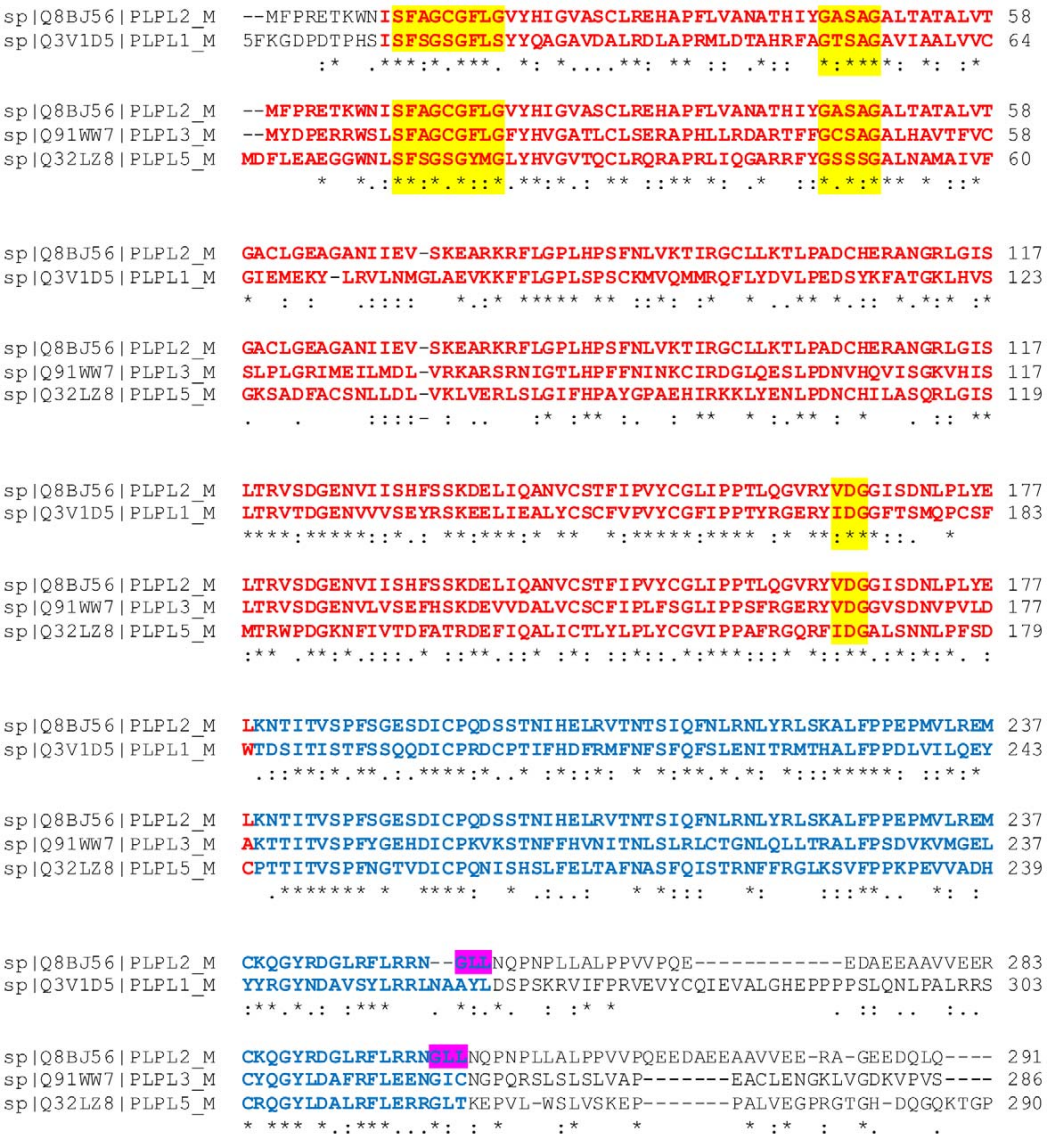
Amino acid sequence alignment of PNPLA family members. The amino acid sequence alignment of mouse PNPLA family members PNPLA1, -2, -3, and -5 shows high sequence conservation at the N-terminal halves of the proteins. Residues at the proposed catalytic sites (oxyanion hole, GXSXG motif, catalytic Asp) are highlighted in yellow. The patatin domain (Ile10-Leu178 for ATGL/PNPLA2) is emphasized in bold red letters. High identity can be also observed in the patatin-related region, up to Leu254 (bold blue).

It is also interesting to note, that high sequence identity of ATGL (PNPLA2) with other members of the PNPLA family (PNPLA1, PNPLA3, PNPLA5) continues further than the usually proposed patatin-domain (residue 10–178 in PNPLA2, highlighted in red bold in Figure 8). Mouse PNPLA2 and PNPLA1 share 66% and 67% similarity within residues 10–178 and 5–254, respectively (numbers refer to PNPLA2). Mouse PNPLA2, PNPLA3, and PNPLA5 shares 63% and 60% similarity within residues 10–178 and 5–254, respectively. Interestingly, sequence alignments display larger gaps and a drop in sequence identity after Leu254 (indicated in pink in Figure 8). This could indicate a boundary from a commonly shared domain and the beginning of a separate domain or a Pro-rich linker region. Only few studies on enzymatic activities for the human orthologs of these enzymes are published and report predominantly TG-hydrolyzing activity in addition to low transacylase and phospholipase A2 activity for PNPLA2; TG-hydrolase, transacylase and modest phospholipase A2 activity for PNPLA3. The biochemical functions of PNPLA1 and PNPLA5 are still elusive. These similar activities could be exerted by the well-aligned regions, whereas the remaining C-terminal residues could modulate enzymatic function and substrate specificities of the full-length proteins.

The experimentally determined requirements for the minimum active domain of mATGL are also supported by the following observations: i) Secondary structure prediction: It is predicted that α and β elements are ranging until residue Ala264 before the start of a long loop region; ii) Sequence conservation across species: Analysis of the amino acid sequences of ATGL orthologs from human, mouse, rat, and *Drosophila melanogaster* (brummer lipase, bmm) shows strong sequence conservation up to residue Leu254. Mouse and rat ATGL show 99% sequence identity within the first 278 amino acids, before the start of an apparent linker region in mouse ATGL (Gln288-Asp295), which seems to be missing in the sequence for rat ATGL. In humans, a highly conserved 253 residue long patatin domain containing protein PNPLA4 (also known as gene sequence-2 (GS2)) has also been described, hinting at a domain boundary in this region (this protein is not present in the mouse PNPLA family); and iii) Expression levels of ATGL variants: shorter variants of ATGL than ATGL254 were either misfolded, unsoluble, or degraded rapidly as deduced from the low concentration of these variants in the soluble fractions of bacterial lysates.

ATGL fragments shorter than ATGL254 failed to exert hydrolytic activity despite the presence of the proposed catalytic residues Ser47 and Asp166. According to our model this could be due to the fact that the protein is not folded properly, e.g. the last β-strand of the central sheet cannot be formed anymore, thus yielding a an inactive protein.

Several mutations in the N-terminal half of human ATGL have been observed in patients suffering from NLSDM [9], [10], [36]. These can also be discussed in view of the homology model of ATGL: A duplication event on the sequence level at the position coding for residue 160 leads to the loss of the catalytic residue Asp166 and a premature stop codon at amino acid 178, which obviously explains the lack of function. Deletion events on the sequence level at positions coding for residues Asn180 or Pro231 lead to the loss of residues forming the two helices on the concave side of the central strand and two β-strands of the central sheet. In full agreement with our *in vitro* data, these truncated proteins are not functional for TG hydrolase activity of ATGL. A single missense mutation (Pro195Leu) is reported to result in an enzymatically completely inactive protein [20]. Pro195 resides in a surface exposed loop and thus could be involved in protein-protein interaction surfaces or influence protein dynamics.

Recently, Duncan *et al.* also tested point mutations of N-terminal glycines (Gly14Ala, Gly16Ala, Gly19Ala) and hydrophobic residues (Phe17Ala, Leu18Ala, Val20Ala). According to our model, this region is involved in forming the oxyanion hole in the active site of ATGL (Figure 7A). The formation of this loop and correct positioning of the backbone amides as H-bond donors could also be facilitated by the NH backbone atoms of the rather small amino acid Ala, used for those point mutations. Thus, the retention of the catalytic activity of some of those constructs could be explained. cPLA_2_ and Pat17 both have an Arg residue (Gly-Gly-X-Arg) after the oxyanion hole, which is thought to stabilize the phosphate moiety of a phospholipid substrate with its side chain guanidinium group. Our 3D model of ATGL harbors hydrophobic amino acids in a partly surface exposed turn and an α helix following the oxyanion hole forming residues. These hydrophobic amino acids might contribute to the specific selectivity of ATGL and/or the correct fold of the protein.

Interestingly, the 3D structures of Pat17 and cPLA_2_ harbor additional residues (Val319-Lys383 and Phe640-Tyr719), which are not included in the sequence alignment used for the model ATGL254. These residues form mainly α-helical elements, which complete the 3D structures (Val319-Lys383 colored yellow for Pat17 in Figure 7C). Despite low sequence identity in this region between Pat17 and mATGL, secondary prediction of residues C-terminal of ATGL254 also predicts α-helices and loop regions. It will be interesting to see from an experimentally determined structure of mATGL whether these additional residues indeed adopt a similar conformation.

In summary our results provide insights into the domain boundaries of the active fragment of ATGL to be at residue Leu254, and restrict the essential interaction region with ATGL's regulators CGI-58 and G0S2. The calculated 3D homology model further supports the notion that the patatin-related region of ATGL does not stop at residue Leu178 but ranges at least until residue Leu254.

## Supporting Information

Table S1 **Primers used for cloning mouse ATGL (full-length, C-terminal truncations and ATGL254-MBP) and G0S2.** Underlined: restriction endonuclease cleavage sites.
(DOC)
