# Supplementary material for: The Minimal Domain of Adipose Triglyceride Lipase (ATGL) Ranges until Leucine 254 and Can Be Activated and Inhibited by CGI-58 and G0S2, Respectively
Source: PLoS One. 2011 Oct 19;6(10):e26349. doi: 10.1371/journal.pone.0026349 (PMC3198459; doi:10.1371/journal.pone.0026349)
Supplement: Table S1 — Primers used for cloning mouse ATGL (full-length, C-terminal truncations and ATGL254-MBP) and G0S2. Underlined: restriction endonuclease cleavage sites. (DOC) [file pone.0026349.s001.doc]

| **Full-length mouse ATGL construct *E. coli*** | | |
| --- | --- | --- |
| mATGL_forward | | 5’-CGAGCACGAGTCGACATCACCATCACCATCACCATGAAAACCTGTATTTTCAGGGCTTCCCGA  GGGAGACCAAGTGGAAC-3’ |
| mATGL_reverse | | 5’-GCAGAACGACGGAAGTCAGCAAGGCGGGAGGCCAGGTGG-3’ |
| **Mouse ATGL C-terminal truncations** | | |
| ATGL400_forward | | 5’-CAGACTGTCTGAGCAGGTGGAATAACGACGTGCCCAGT-3’ |
| ATGL400_reverse | | 5’-ACTGGGCACGTCGTTATTCCACCTGCTCAGACAGTCTG-3’ |
| ATGL364_forward | | 5’-GTGGCTGCCTGATGTCCCTGAAGATTAACGGTGGATGAAAGA-3’ |
| ATGL364_reverse | | 5’-TCTTTCATCCACCGTTAATCTTCAGGGACATCAGGCAGCCAC-3’ |
| ATGL295_forward | | 5’-GATCAATTGCAGCCTTATAGAAAATAGCGAATTCTAGAGCACCTGC-3’ |
| ATGL295_reverse | | 5’-GCAGGTGCTCTAGAATTCGCTTATTTTCTATAAGGCTGCAATTGATC-3’ |
| ATGL288_forward | | 5’-GGGCTGGAGAGGAGGATTAATTGCAGCCTTATAGA-3’ |
| ATGL288_reverse | | 5’-TCTATAAGGCTGCAATTAATCCTCCTCTCCAGCCC-3’ |
| ATGL279_forward | | 5‘-GAGGAAGCTGCTGTGTAGGAGGAGAGGGCTGG-3‘ |
| ATGL279_reverse | | 5’-CCAGCCCTCTCCTCCTACACACAGCAGCTTCCTC-3’ |
| ATGL272_forward | | 5’-CTACTGAACCAACCCAACCCTTAACTGGCACTGCCC-3’ |
| ATGL272_reverse | | 5’-GGGCAGTGCCAGTTAAGGGTTGGGTTGGTTCAGTAG-3’ |
| ATGL260_forward | | 5’-CTACTGAACCAACCCAACCCTTAACTGGCACTGCCC-3’ |
| ATGL260_reverse | | 5’-GGGCAGTGCCAGTTAAGGGTTGGGTTGGTTCAGTAG-3’ |
| ATGL257_forward | | 5’-CCTTAGGAGGAATGGCCTACTGAACCAATAAAACCCTTTGCTGGCA-3’ |
| ATGL257_reverse | | 5’-TGCCAGCAAAGGGTTTTATTGGTTCAGTAGGCCATTCCTCCTAAGG-3’ |
| ATGL255_forward | | 5’-GGAGGAATGGCCTACTGTAACAACCCAACCCTTTGCT-3’ |
| ATGL255_reverse | | 5’-AGCAAAGGGTTGGGTTGTTACAGTAGGCCATTCCTCC-3’ |
| ATGL254_forward | | 5’-CTTCGATTCCTTAGGAGGAATGGCCTATAAAACCAACCCAACCCTTT-3’ |
| ATGL254_reverse | | 5’-AAAGGGTTGGGTTGGTTTTATAGGCCATTCCTCCTAAGGAATCGAAG-3’ |
| ATGL253_forward | | 5’-CCTTAGGAGGAATGGCTGACTGAACCAACCCAACC-3’ |
| ATGL253_reverse | | 5’-GGTTGGGTTGGTTCAGTCAGCCATTCCTCCTAA-3’ |
| ATGL252_forward | | 5’-ACTTCGATTCCTTAGGAGGAATTAACTACTGAACCAACCCAACCCT-3’ |
| ATGL252_reverse | | 5’-AGGGTTGGGTTGGTTCAGTAGTTAATTCCTCCTAAGGAATCGAAGT-3’ |
| ATGL245_forward | | 5’-GTGCAAACAGGGCTACAGAGATGGATAACGATTCCTTAGGAGGAAT-3’ |
| ATGL245_reverse | | 5’-ATTCCTCCTAAGGAATCGTTATCCATCTCTGTAGCCCTGTTTGCAC-3’ |
| ATGL235_forward | | 5’-GCCCATGGTCCTCCGATAAATGTGCAAACAGGGCT-3’ |
| ATGL235_reverse | | 5’-AGCCCTGTTTGCACATTTATCGGAGGACCATGGGC-3’ |
| **Mouse ATGL254-MBP** | | |
| 254_MBP_forward | 5’-CGATAGCGGCCGCAATGTTCCCGAGGG-3’ | |
| 254_MBP_reverse | 5’-GGACTGGATCCCTATAGGCCATTCCTCC-3’ | |
| **Mouse G0S2 in pCOLD** | | |
| mG0_forward | 5’-AGCTACGCATATGGAAAGTGTGCAGGAGC-3’ | |
| mG0_reverse | 5’-CCTGACAAGCTTTTAAGAGGCGTGC-3’ | |

**Table S1: Primers used for cloning mouse ATGL (full-length, C-terminal truncations and ATGL254-MBP) and G0S2.**

Underlined: restriction endonuclease cleavage sites.
